# Canadian Midwives’ Experiences with Nutrition in Their Training and Practice: A Cross‐Sectional Study

**DOI:** 10.1111/jmwh.13665

**Published:** 2024-08-05

**Authors:** Jordyn Butler, Yvana Sawaya, Jamie A. Seabrook, Janet Madill, Jasna Twynstra

**Affiliations:** ^1^ School of Food and Nutritional Sciences Brescia University College London Ontario Canada; ^2^ Department of Epidemiology and Biostatistics Western University London Ontario Canada; ^3^ Human Environments Analysis Laboratory Western University London Ontario Canada; ^4^ Department of Paediatrics Western University London Ontario Canada; ^5^ Children's Health Research Institute London Ontario Canada; ^6^ Lawson Health Research Institute London Ontario Canada

**Keywords:** midwifery education, nutrition, patient education

## Abstract

**Introduction:**

Midwives are primary prenatal care providers well‐positioned to offer nutrition advice to pregnant individuals; however, no Canadian study has assessed midwives’ experience with nutrition education. The objective of this study was to investigate Canadian midwives’ experiences with nutrition in their practice, their level of nutrition education, and their recommendations on select nutrition topics.

**Methods:**

This cross‐sectional study used an anonymous online survey consisting of 4 sections: demographics, opinions on the importance of nutrition, nutrition recommendations for pregnancy, and nutrition topics that midwives would like more information on. Responses were recorded using Likert‐type scales, multiple choice, or open‐ended questions. Eligible participants, registered Canadian midwives, were recruited through advertisements in e‐newsletters via national and provincial midwifery associations, social media posts, and emails to midwifery clinics. An independent samples *t* test compared differences in means for continuous outcomes, the χ^2^ test compared categorical variables, and the Mann‐Whitney *U* test compared ordinal variables. A *P* < .05 was considered statistically significant.

**Results:**

In total, 161 midwives completed the online survey. Most midwives (92.5%) indicated that nutrition for pregnancy was important, and 83.2% believed their role in providing nutrition information to pregnant women was important. Almost two‐thirds (63.8%) of midwives received nutrition education. Comfort levels were highest (median = 4) when providing nutrition advice on healthy eating, weight gain, Listeria, anemia, heartburn, safe food handling, nutrition for breastfeeding, and weight gain for women with obesity. Almost all the midwives (99.4%) had provided nutrition information to pregnant women, and 85.2% of their recommendations aligned with Canadian guidelines and literature.

**Discussion:**

Canadian midwives valued the importance of nutrition during pregnancy and their role in providing nutrition information to pregnant women. The level of comfort in advising on nutrition ranged from uncomfortable to very comfortable depending on the topic, and most (85.2%) of their advice aligned with Canadian guidelines and relevant literature.

## INTRODUCTION

Maternal nutrition is an important consideration for pregnancy. Healthy nutrition during pregnancy can decrease the risk of gestational diabetes, hypertensive disorders, and preterm birth.^1^, ^2^, ^3^ In contrast, poor maternal nutrition is associated with low birth weight, preterm birth, macrosomia, and an increased risk for the infant to develop chronic diseases later in life, such as cardiovascular diseases and diabetes.^4^, ^5^, ^6^, ^7^, ^8^

Continuing education (CE) is available for this article. To obtain CE online, please visit http://www.jmwhce.org. A CE form that includes the test questions is available in the print edition of this issue.

Nutrition advice from health care providers (HCPs) can positively influence maternal and infant outcomes. Nutrition counseling of pregnant women has been reported to improve gestational weight gain; reduce rates of adverse birth outcomes such as low birth weight, large for gestational age, and preterm birth; and decrease the risk of gestational diabetes.^9^, ^10^, ^11^ Furthermore, dietary interventions aimed at optimizing gestational weight gain were more impactful when they were delivered by HCPs and when they were tailored to the individual compared with interventions not delivered by HCPs or those delivered in a group setting.^12^ As nutrition advice and interventions are correlated with optimal pregnancy outcomes, all pregnant people should receive nutrition counseling from their HCPs.

**QUICK POINTS**

✦ Approximately two‐thirds (63.8%) of Canadian midwives surveyed receive nutrition training in their education.
✦ Almost all (99.4%) Canadian midwives surveyed provide prenatal nutrition information to their clients.
✦ Midwives feel comfortable providing nutrition information.
✦ Nutrition education does not correlate with nutrition recommendations midwives provide.
✦ Midwives’ nutrition recommendations largely align with Canadian guidelines and relevant literature (85.2%).

In Canada, general practitioners, obstetricians, and midwives are the most common primary HCPs who provide prenatal care for pregnant women.^13^ Midwifery services are increasing in Canada, as the number of practicing midwives grew from 1690 in 2017 to 1892 in 2021, whereas midwifery‐attended births increased from 3% in 2001 to 13% in 2021, with similar increasing trends reported in the United States.^14^, ^15^, ^16^ Although studies have explored midwives’ provision of nutrition advice, no Canadian study has assessed midwives’ experience with nutrition education.^17^, ^18^, ^19^ It has been reported that Canadian midwives offer limited counseling on recommended weight gain,^18^ are aware of the benefits of vitamin D supplementation, and are aware that iron deficiency is a problem.^17^ In comparison, research from Australia, New Zealand, and Europe suggests that midwives receive limited nutrition education but believe that nutrition counseling is an important aspect of their role in patient care, have a positive attitude toward nutrition and its importance during pregnancy, and provide nutrition advice to their pregnant clients.^20^, ^21^, ^22^, ^23^, ^24^ Limited research exists from the United States, with studies suggesting that midwives offer nutrition advice, but the information does not always align with recommendations.^25^, ^26^ In addition, The American College of Nurse‐Midwives asserts that there are nutrition components missing from midwifery education.^27^

Given the growth of the midwifery profession in Canada, more research needs to focus on Canadian midwives and their experiences with nutrition in their education and practice. Therefore, the objective of this study was to investigate Canadian midwives’ experiences with nutrition in their practice, their level of education in providing nutrition information, and their recommendations on select nutrition topics.

## METHODS

This cross‐sectional study was completed using an anonymous, web‐based survey. A convenience sample of registered midwives in Canada was collected via 4 recruitment strategies: (1) a recruitment message included in the weekly e‐newsletter by the Canadian Association of Midwives; (2) social media (Twitter and Facebook) recruitment through the personal pages of the research team; (3) distribution of the recruitment message by select provincial and territorial midwifery associations; and (4) email recruitment to publicly available email addresses of midwifery clinics in Canada. The survey was available to 1909 registered midwives^28^ from June to November 2019, and consent was implied when midwives completed the survey. The Health Sciences Research Ethics Board at Western University approved the study (112013). The study is reported in accordance with the Strengthening the Reporting of Observational Studies in Epidemiology guidelines for cross‐sectional studies (Supporting Information: Appendix S1).

### Survey Development

The survey was created using the online platform Qualtrics. The original survey was developed by the researchers after consulting the literature focused on midwives and their experiences with nutrition in their education and practice.^20^, ^21^ The final survey was reviewed by a registered dietitian (J.M.) and was pilot‐tested by obstetricians‐gynecologists (n = 5). The survey was divided into 4 sections that focused on demographics, opinions on the importance of nutrition, nutrition recommendations for pregnancy, and nutrition topics that midwives would like more information on.

### Demographics

This section of the survey asked close‐ended questions regarding age, years of experience in midwifery, and area of practice. Midwives were also asked if they received their midwifery education in Canada and if nutrition education was incorporated during and/or after their midwifery education program.

### Thoughts and Opinions on the Importance of Nutrition

Using a 5‐point Likert‐type scale, from 1 (very unimportant) to 5 (very important), midwives were asked to rate the importance of healthy nutrition during pregnancy and how central they believe a midwife's role is in providing nutrition information to pregnant women. During data analysis, these 5‐point Likert‐type scales were reduced to 3 categories: important (including options 4 and 5), neither important nor unimportant (including option 3), and unimportant (including options 1 and 2) due to a small count of responses for options 1 and 5.

Midwives were also asked when and how they provide nutrition information to their clients, which information sources they use to enhance their nutrition knowledge, and about their experiences with using registered dietitian (RD) services or referrals to other HCPs. Finally, comfort on advising and frequency of encountering specific nutrition topics were rated using Likert‐type scales.

### Nutrition Recommendations for Pregnancy

Seven questions were asked about midwives’ nutrition recommendations for pregnancy. Topics focused on methods to reduce nausea and vomiting, nutrients of concern for a specialty diet, foods to avoid due to the risk of Listeria, recommended foods to manage constipation, prepregnancy body mass index (BMI) ranges, folic acid supplementation recommendations, and safety regarding herbal supplements. Questions were either multiple choice or broken into yes or no categories for each response option. All questions, except for one question that was eliminated from the analysis, had at least one answer that aligned with current Canadian guidelines for pregnancy and relevant literature.^29^, ^30^, ^31^, ^32^, ^33^, ^34^

### Nutrition Resources

The fourth section of the survey included an open‐ended question that allowed midwives to identify any nutrition topics they would like more information on.

### Statistical Analysis

Data were analyzed using IBM SPSS Statistics, version 27. Descriptive statistics (frequencies and percentages) were used to describe the study population and responses to categorical questions (eg, recommendations for specific nutrition topics). Age was reported as a mean with SD, whereas Likert‐type scale responses were summarized using medians. A χ^2^ test was used to test for associations between categorical variables. An independent samples *t* test compared differences in means for continuous outcomes, whereas the Mann‐Whitney *U* test compared differences in ordinal variables between 2 groups. To measure the strength and direction of an association between 2 ordinal variables, the Spearman rank correlation coefficient was used, where coefficients (+/−) between 0.10 and 0.29 were considered a small correlation, 0.30 and 0.49 a medium correlation, and 0.50 or above a large correlation.^35^ A value of *P* < .05 was considered statistically significant.

For nutrition recommendation questions, the data were expressed as a mean score calculated as the percentage of responses that aligned with the guidelines and literature based on the number of questions answered. A total of 45 out of 161 midwives did not respond to all questions. Therefore, a sensitivity analysis was conducted that removed all respondents with incomplete answers through listwise deletion.^36^ No differences were found between the calculated mean score from all answered questions and listwise deletion analysis. Thus, the mean calculated score data are presented.

## RESULTS

### Demographics

Out of a total of 1909 practicing midwives,^28^ 161 midwives completed the survey, with a response rate of 8.4%. The sociodemographic and education characteristics of the sample can be found in Table 1. The mean (SD) age of the midwives was 40.3 (9.9) years. Most midwives were practicing in Ontario (54.4%), British Columbia (25.6%), and the Prairies (17.5%). Three‐quarters (75.0%) of the midwives practiced in an urban or suburban setting. Seventy‐eight percent of midwives received their midwifery education in Canada. Almost two‐thirds (63.8%) of midwives received nutrition education, of whom 58.1% received it during their midwifery program and 17.4% after. The most frequent method to deliver nutrition education during the midwifery education program was through integration into one or more courses (93.3%). Among those who received nutrition education after their midwifery education program, the most frequent education method was through workshops (68.0%).

**Table 1:** Sociodemographic and Education Characteristics of Midwives (N = 161)

Characteristics	Value
**Age, mean (SD), y**	40.3 (9.9)
**Years of experience, n/total (%)**	
<2	30/161 (18.6)
2‐5	42/161 (26.1)
6‐10	36/161 (22.4)
11‐20	36/161 (22.4)
>20	17/161 (10.6)
**Province or territory of practice, n/total (%)**	
British Columbia	41/160 (25.6)
Nova Scotia	1/160 (0.6)
Nunavut	3/160 (1.9)
Ontario	87/160 (54.4)
Prairies	28/160 (17.5)
**Setting of practice, n/total (%)**	
Urban/suburban	120/160 (75.0)
Rural/remote	40/160 (25.0)
**Obtained midwifery education in Canada, n/total (%)**	
Yes	126/161 (78.3)
No	35/161 (21.7)
**Nutrition education received, n/total (%)**	
During or after midwifery program	102/160 (63.8)
During and after midwifery program	19/160 (11.9)
Only during midwifery program	93/160 (58.1)
Only after midwifery program	28/161 (17.4)
Did not receive nutrition education	58/160 (36.3)
**Method of education *during* midwifery education program, n/total (%)**	
As a required nutrition course	29/79 (36.7)
As an elective nutrition course	9/71 (12.7)
Nutrition information was integrated into one or more courses	83/89 (93.3)
During practicum placement	58/79 (73.4)
**Method of education *after* midwifery education program, n/total (%)**	
In clinic/hospital	6/22 (27.3)
Online courses	13/23 (56.5)
Workshops	17/25 (68.0)
Other (please specify)^a^	7/16 (43.8)

a Other methods of education midwives received after their Midwifery Education Program included self‐directed learning and referencing current professional guidelines.

### Thoughts and Opinions on the Importance of Nutrition

Most (92.5%) midwives thought nutrition for pregnancy was important, and 83.2% believed their role in providing nutrition information to pregnant women was also important. Almost all (99.4%) midwives indicated that they had provided nutrition information to pregnant women. The most common occasions for providing nutrition advice were at the woman's request (100%), in case of a medical condition (96.8%), and at the first antenatal visit (96.2%). Providing verbal advice to pregnant women was the most frequent (99.4%) method to deliver nutrition information, whereas directing clients to mobile applications was the least selected method (18.4%). Approximately three‐quarters (73.9%) of midwives had access to RD services, and 64.0% referred clients to an RD while 36.0% of midwives had never referred clients to an RD. The main barrier to making a referral was because their client could not afford dietitian services (56.2%). The midwives often referred to clinical practice guidelines, other HCPs, government or official websites, and research articles to enhance their knowledge (median, sometimes) (Table 2).

**Table 2:** Midwives’ Attitudes Toward the Importance of Nutrition During Pregnancy and Their Experience in Providing Nutrition Advice, as Well as Accessing Dietitian Services and Materials Used to Enhance Their Own Nutrition Knowledge (N = 161)

Question	Value
**Rate the importance of healthy nutrition during pregnancy, n (%)**	
Unimportant	3 (1.9)
Neither important nor unimportant	9 (5.6)
Important	149 (92.5)
**Rate the importance ofa midwife's role is in providing nutrition information to pregnant women, n (%)**	
Unimportant	2 (1.2)
Neither important nor unimportant	25 (15.5)
Important	134 (83.2)
**Provided nutrition information to pregnant women, n/total (%)**	
Yes	160/161 (99.4)
No	1/161 (0.6)
**Occasions when nutrition information was provided: n/total** ^a^ **(%)**	
First antenatal visit	152/158 (96.2)
Every antenatal visit	26/137 (19.0)
In case of a medical condition	153/158 (96.8)
At the woman's request	159/159 (100)
**Provided nutrition advice in the following ways: n/total (%)**	
Pamphlet/booklet	94/154 (61.0)
Verbally	159/160 (99.4)
Directed to website	112/153 (73.2)
Directed to mobile applications	25/136 (18.4)
Other (please specify)^b^	33/95 (34.7)
**Access to registered dietitian services, n/total (%)**	
Yes	119/161 (73.9)
No	42/162 (26.1)
**Made referrals to a registered dietitian, n/total (%)**	
Yes	103/161 (64.0)
No	58/161 (36.0)
**Main barriers for making referrals to a registered dietitian, n/total (%)**	
I do not have access to a dietitian	38/129 (29.5)
My clients can't afford a dietitian	77/137 (56.2)
Clients are not interested in receiving nutritional information	59/136 (43.4)
Other (please specify) ^c^	37/81 (45.7)
**Frequency the following sources were used to enhance personal nutrition knowledge, median** ^d^	
Other health professionals	Sometimes
Government or official websites	Sometimes
Research articles	Sometimes
Textbooks	Rarely
Social media	Rarely
Media (TV, newspaper, magazines)	Rarely
Clinical practice guidelines (eg, SOGC)	Sometimes
Other (please specify)^e^	Never

Abbreviation: SOGC, Society of Obstetricians and Gynecologists of Canada.

a n/total n = number of midwives who selected *yes* out of the total number of respondents.

b Other occasions include books, Canada's Food Guide, and research literature.

c Other barriers included appointments only available during work hours, confusion surrounding reason for referral, do not trust alignment with midwifery model of care/midwives do not agree with registered dietitian [RD] advice, RD availability is limited.

d Median reported of the 4‐point Likert scale (never, rarely, sometimes, frequently).

e Other information sources included professional development courses, conferences, and Association of Ontario Midwives practice guidelines.

Overall, midwives felt most comfortable providing advice on healthy eating, weight gain, Listeria, anemia, heartburn, safe food handling, nutrition for breastfeeding, and weight gain for obesity (median, 4). Similarly, they most frequently encountered topics of healthy eating, weight gain, anemia, heartburn, and nutrition for breastfeeding (median, 4). The least encountered topics were vegan and ketogenic diets, Listeria, and nutrition for women of different ethnic origins (median, 2). The comfort levels of midwives when providing advice on nutrition‐related topics were significantly correlated (*P* < .05) with the frequency with which they encountered nutrition topics, except for anemia. Furthermore, weak to moderate correlations were found between midwives’ comfort level in providing nutrition information and the frequency with which they encountered nutrition‐related topics (Table 3).

**Table 3:** Correlations Between Frequency of Encounter and Comfort Level in Providing Nutrition Advice on Nutrition‐Related Topics

Nutrition‐Related Topic	Frequency Median^a^	Comfort Median^b^	Spearman Correlation Coefficient	*P* value
Healthy eating	4.0	4.0	0.26	.001
Weight gain	4.0	4.0	0.18	.02
Vegetarian diet	3.0	3.5	0.34	<.001
Vegan diet	2.0	3.0	0.23	<.01
Ketogenic diet	2.0	2.0	0.46	<.001
Listeria	2.0	4.0	0.41	<.001
Anemia	4.0	4.0	0.08	.31
Heartburn	4.0	4.0	0.17	.03
Safe food handling	3.0	4.0	0.34	<.001
Nutrition for breastfeeding	4.0	4.0	0.17	.03
Nutrition for women with gestational diabetes	3.0	3.0	0.32	<.001
Weight gain for women with obesity	3.0	4.0	0.25	.001
Herbal supplements	3.0	3.0	0.47	<.001
Nutrition for women of different ethnic origins	2.0	3.0	0.43	<.001

a 4‐point Likert scale (never encounter = 1, rarely encounter = 2, sometimes encounter = 3, frequently encounter = 4).

b 5‐point Likert‐type scale 1 (very uncomfortable) to 5 (very comfortable).

### Nutrition for Pregnancy

When midwives were asked to identify the appropriate BMI for a woman considered overweight, more than half (59.7%) of the midwives selected 25.0‐29.9 kg/m^2^. The majority of midwives (88.2%) recommended that women begin taking a folic acid supplement at least 3 months before pregnancy. Almost two‐thirds (64.6%) of midwives indicated they would not advise their clients that herbal supplements are safe for pregnancy. To minimize the effect of nausea during pregnancy, all midwives (100%) indicated they would recommend their clients to eat small frequent meals, and 98.1% recommended avoiding having an empty stomach. To manage constipation during pregnancy, all midwives (100%) recommended their clients to consume fruits and vegetables, and 98.1% recommended water and juice consumption. When midwives were asked to identify foods to avoid during pregnancy due to the risk of Listeria, most recommended their clients avoid deli meats (88.7%), soft cheeses (80.5%), and raw fish sushi (74.7%). The nutrients midwives would be most concerned about if their client followed a vegan diet were omega‐3 fatty acids (83.8%), followed by vitamin D (65.0%). The mean (SD) percentage of all responses that aligned with Canadian guidelines was 85.2% (9.4%). Additionally, there was no relationship between midwives who received or did not receive nutrition education when providing nutrition recommendations to pregnant women that align with Health Canada's pregnancy guidelines and relevant literature (*P* = 0.75) (Table 4).^29^, ^30^, ^31^, ^32^, ^33^, ^34^

**Table 4:** Summary of Midwives’ Nutrition Recommendations for Pregnancy

Question	Value
**Advice for minimizing the effect of nausea, n/total** ^a^ **(%)**	
Drink plenty of fluids with meals	19/153 (12.4)
Avoid having an empty stomach^b^	158/161 (98.1)
Eat large quantities of food at meal times	2/155 (1.3)
Eat small frequent meals^b^	161/161 (100)
**Prepregnancy BMI range considered as overweight, n/total (%)**	
18.5‐24.9	1/159 (0.6)
25.0‐29.9^b^	95/159 (59.7)
30.0‐34.9	59/159 (37.1)
I do not know	4/159 (2.5)
**Ideal recommendation on when to begin taking a folic acid supplement, n/total (%)**	
At least 3 mo before pregnancy^b^	142/161 (88.2)
At least 1 mo before pregnancy	16/161 (9.9)
After confirming one is pregnant	3/161 (1.9)
Before first trimester is complete	0/161 (0)
**Nutrients of concern for clients following a vegan diet, n/total (%)**	
Vitamin D^b^	91/140 (65.0)
Zinc	47/134 (35.1)
Vitamin A	39/133 (29.3)
Omega‐3 fatty acids^b^	124/148 (83.8)
**Recommended foods to avoid due to risk of Listeria, n/total (%)**	
Soft cheeses^b^	128/159 (80.5)
Hard cheeses	8/132 (6.1)
Deli meats^b^	141/159 (88.7)
Raw fish sushi^b^	118/158 (74.7)
**Foods recommended for managing constipation, n/total (%)**	
Water or juice^b^	157/160 (98.1)
Dairy foods	1/142 (0.7)
Fruits and vegetables^b^	161/161 (100)
Meats	1/141 (0.7)
**Are herbal supplements safe for pregnancy? n/total (%)**	
Yes	57/161 (35.4)
No^b^	104/161 (64.6)
**Responses aligning with Canadian guidelines, mean (SD)**	
All midwives	85.2 (9.4)
Midwives with nutrition education	85.5 (9.5)^c^
Midwives without nutrition education	85.0 (9.5)

a n/total n = number of midwives who selected *yes* out of the total number of respondents.

b Indicates the answer option that aligns with Health Canada's pregnancy guidelines and relevant literature.

c Compared with midwives who received nutrition education using an independent samples *t* test (*P* = 0.75).

## DISCUSSION

The main findings of this study are that Canadian midwives value the importance of healthy nutrition during pregnancy and their role in providing nutrition information to pregnant women. Two‐thirds of midwives received nutrition education, and almost all (99.4%) provided nutrition information to pregnant women and felt comfortable providing nutrition advice on nutrition‐related topics such as anemia, healthy eating, safe food handling, heartburn, and nutrition for breastfeeding. Most (85.2%) midwives’ nutrition recommendations aligned with Health Canada's pregnancy guidelines and relevant literature, and there was no correlation between the nutrition education received and the recommendations they provided.

The findings of this study are supported by other Canadian studies, which reported that midwives provide the most nutrition advice to their clients compared with general practitioners and family doctors, obstetricians, nurse practitioners, and registered nurses.^19^, ^37^ The positive outlook of Canadian midwives on nutrition for pregnancy and their role in providing this advice is consistent with studies from Australia and New Zealand.^20^, ^21^ Furthermore, the comfort level of midwives in providing nutrition advice on nutrition‐related topics is similar to a study from New Zealand, which showed that midwives were confident in advising on nausea, heartburn, anemia, nutrition for breastfeeding, weight gain, and Listeria,^21^ and studies from England^38^ and Australia,^20^ which showed that midwives were less confident discussing general or specific nutrition topics and complex nutrition issues with pregnant women. To the authors’ knowledge, no study from the United States reports on midwives’ knowledge and comfort levels when providing advice on general nutrition for pregnancy.

The significant relationship between frequency of encounter and comfort levels for all but one nutrition topic included in this study suggests the possibility that increased exposure to specific nutrition topics may increase comfort levels when providing advice on these topics. For example, midwives may feel more comfortable providing information about anemia because they frequently encounter this topic and because anemia is highly prevalent in the population, with approximately 41.8% of women experiencing anemia worldwide during pregnancy.^39^ Similarly, the ketogenic diet, a low carbohydrate diet, has gained popularity over the past decade,^40^ but only 0.4% of Canadian women follow this diet during pregnancy.^41^ Therefore, the midwives’ rare encounters with it may explain their low levels of comfort when providing information about this topic. Another possibility for limited comfort levels could be the lack of guidance for specific nutrition topics, like herbal supplements. For example, there are limited studies on herbal supplements and their safety during pregnancy,^42^ and Health Canada indicates that some herbal teas are not safe for pregnancy, whereas others are only safe in moderation.^30^ The complexity of the safety of herbal supplements for pregnancy may be contributing to the lower level of comfort when advising on this topic.

Nutrition education programs for midwives have been suggested to increase nutrition knowledge.^43^ However, given that approximately one‐third (36.3%) of Canadian midwives did not receive nutrition training during their education, this suggests that nutrition education may not be emphasized in the Canadian curriculum for midwifery. From a global perspective, midwifery education in the United States, United Kingdom, Australia, and New Zealand is reported to have significant similarities; however, the curriculum has not been directly compared.^44^ Nevertheless, midwives in the current study indicated that they consult credible sources of information, such as clinical practice guidelines, research articles, and government websites, as well as other HCPs, to gain more knowledge on nutrition topics. Because 85.2% of their nutrition recommendations are in alignment with guidelines, this suggests that midwives have adequate knowledge of nutrition. Additionally, Canadian midwives use information delivery techniques that pregnant women prefer, including verbal conversation from their HCP, which could have positive impacts on nutritional intake during pregnancy.^45^

The most nutrition‐trained HCPs are RDs.^46^ Although studies on the effect of RD involvement in prenatal care show inconsistent results,^47^, ^48^, ^49^ a recent systematic review suggests that RD involvement in prenatal care may improve adverse birth outcomes, such as low birth weight and preterm birth.^10^ However, midwives in this study identified barriers to making referrals to RDs, with the main barrier being that the clients cannot afford them. In Canada, RDs are not covered by many provincial or territorial health insurance plans, and consultation with the RD must be paid for by employee or private health insurance benefits or out of pocket by the client, ultimately limiting accessibility to RD services for some individuals.^50^ Nevertheless, the reported high accessibility and referral to RDs by Canadian midwives indicates that midwives are aware of RDs as nutrition experts.

### Strengths and Limitations

This is the first Canadian study to explore midwives’ experience with nutrition advice for pregnancy. Also, midwives were practicing in several different provinces and geographical settings in our sample, increasing the generalizability of the results. This study, however, does have some limitations. The study is not generalizable to all Canadian midwives, as there was a low response rate and no representation of midwives practicing in certain provinces (eg, Quebec). Additionally, a convenience sample means that midwives who completed the survey might have been more interested, comfortable, and educated about nutrition than midwives who did not participate in the study. Lastly, limited nutrition topics were discussed in the survey, and other nutrition topics pertaining to pregnancy (eg, appropriate gestational weight gain for prepregnancy BMI, caffeine intake, and micronutrient intakes) may offer additional understanding of midwives’ experience with nutrition.

## CONCLUSION

Canadian midwives value the importance of nutrition during pregnancy and their role in providing nutrition information to pregnant women. Furthermore, they provide recommendations that adhere to Canadian guidelines and feel comfortable providing nutrition information on some nutrition‐related topics. Future research should focus on the extent of nutrition education that midwives receive, as well as specific nutrition topics that pregnant people are interested in, so that midwives can be better prepared to care for their clients' nutrition queries.

## CONFLICT OF INTEREST

The authors have no conflicts of interest to disclose.

## Supporting information


**Appendix S1**. Strengthening the Reporting of Observational Studies in Epidemiology (STROBE) checklist
